# Antioxidant functionalized double-net/TA dynamic hydrogel promotes cartilage regeneration through stabilization of chondrocyte phenotype

**DOI:** 10.1016/j.mtbio.2025.102203

**Published:** 2025-08-16

**Authors:** Xu Wu, Honglei Wang, Chenlong Li, Yaying Zhu, Qixuan Wang, Tianyu Zhang, Yaoyao Fu, Aijuan He

**Affiliations:** a Department of Facial Plastic and Reconstructive Surgery, Eye and ENT Hospital, Fudan University, Shanghai, China; b ENT Institute, Eye and ENT Hospital, Fudan University, Shanghai, China; c NHC Key Laboratory of Hearing Medicine (Fudan University), Shanghai, China

**Keywords:** Cartilage regeneration, Chondrocyte dedifferentiation, Single cell sequencing, Double-net hydrogel, Tannin acid

## Abstract

Cartilage defects, whether congenital or acquired, are highly prevalent in clinical practice. Tissue engineering offers a promising strategy for cartilage regeneration; however, the loss of chondrocyte phenotype during *in vitro* expansion remains a major barrier to the clinical translation of chondrocyte-based engineered cartilage. Emerging evidence has highlighted that alterations in chondrocyte metabolic states can profoundly impact their phenotypic stability. Nonetheless, how metabolic patterns shift during *in vitro* expansion, and whether metabolic modulation can stabilize the chondrocyte phenotype, remain insufficiently explored.

To address these questions, we first utilized single-cell RNA sequencing combined with bulk transcriptomic analysis to profile the metabolic reprogramming of chondrocytes during *in vitro* expansion. Our findings revealed a distinct shift from glycolytic metabolism toward oxidative phosphorylation dominance. Based on this insight, we engineered a DN (double-net) hydrogel scaffold composed of collagen, PEG (polyethylene glycol), and CNF (nanocellulose). To endow the scaffold with antioxidant functionality, TA (tannic acid) was incorporated by hydrogen bonding to the CNF network, forming an antioxidant DN-TA hydrogel system.

To evaluate whether attenuating aerobic metabolism could preserve chondrocyte phenotype, P3 (passage 3) chondrocytes were cultured within the hydrogel scaffold *in vitro* and then implanted subcutaneously into nude mice. The DN-TA hydrogel effectively preserved the chondrocyte phenotype by activating HIF-1 signaling pathway and reducing ROS (reactive oxygen species).

Furthermore, after 8/12 weeks of subcutaneous implantation, the DN-TA scaffold significantly enhanced *in vivo* cartilage regeneration, as evidenced by increased extracellular matrix deposition and more mature cartilage formation. Collectively, our study demonstrates that reducing aerobic metabolism helps stabilize the chondrocyte phenotype and promotes functional cartilage regeneration. These findings offer novel insights for optimizing cartilage tissue engineering strategies through metabolic modulation.

## Graphical abstract

**Figure:**
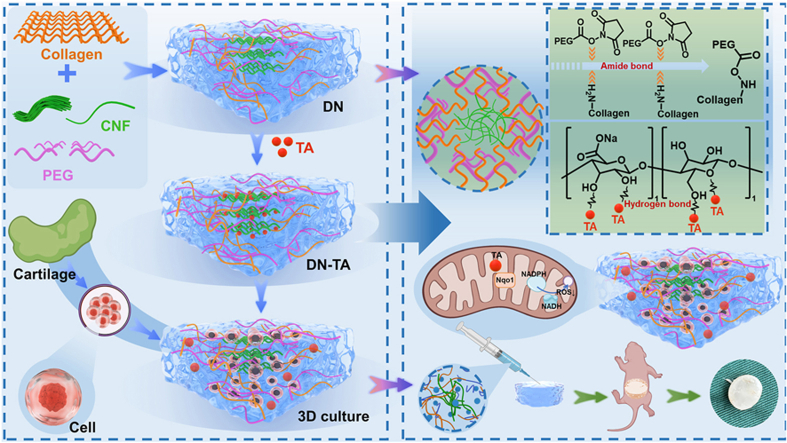
Antioxidant Functionalized Double-Net/TA Dynamic Hydrogel for Cartilage Regeneration.

## 1 Introduction

Microtia is one of the most common craniofacial congenital anomalies [1,2], for which autologous costal cartilage remains the gold standard material for fabricating a three-dimensional auricular framework in reconstructive surgery. However, harvesting costal cartilage is associated with notable drawbacks, including donor-site morbidity and the risk of thoracic deformities. These limitations have prompted an urgent need to identify ideal cartilage substitutes.

Tissue engineering, an interdisciplinary field that integrates life sciences, chemistry, and materials science, has emerged as a promising strategy for cartilage regeneration. In recent years, substantial progress has been made in both preclinical and clinical studies [[1], [2], [3]]. Nevertheless, some investigations have reported that engineered cartilage tends to undergo ossification or fibrosis following *in vivo* implantation, ultimately leading to the failure of cartilage regeneration [4].Early research primarily attributed the failure of tissue-engineered cartilage to chondrocyte dedifferentiation during *in vitro* expansion [[5], [6], [7]]. Our previous work demonstrated that serial *in vitro* expansion of chondrocytes leads to downregulation of ECM (extracellular matrix) markers and a concomitant loss of chondrogenic capacity [5,8,9]. Consequently, many efforts were made to suppress dedifferentiation in hopes of improving cartilage regeneration outcomes, yet these attempts yielded limited success. With the advances of high-throughput sequencing, accumulating evidence now indicates that chondrocytes not only dedifferentiate but also acquire osteogenic fibrotic and phenotypes during *in vitro* culture [10,11].Thus, chondrocyte phenotypic instability is a fundamental barrier to effective cartilage regeneration. Strategies aimed at maintaining chondrocyte phenotype during *in vitro* culture are therefore critical for the successful clinical translation of tissue-engineered cartilage.

Numerous studies have demonstrated that alterations in chondrocyte metabolic states can profoundly influence their phenotypic stability [12,13]. Under physiological conditions, chondrocytes primarily generate energy through glycolysis. This metabolic preference arises from the avascular nature of cartilage tissue, which results in limited oxygen availability and compels chondrocytes to rely predominantly on anaerobic glycolysis for ATP production—even under normoxic conditions [14,15]. This hypoxic adaptation confers an advantage by minimizing the risk of reactive ROS (oxygen species) mediated cellular damage [16].

However, upon isolation and subsequent *in vitro* expansion under atmospheric oxygen levels, this physiologically hypoxic niche is disrupted, leading to substantial metabolic reprogramming. Previous studies reported that murine articular chondrocytes undergo metabolic inversion and mitochondrial dysfunction during *in vitro* expansion [13]. Moreover, several studies have shown that dysregulated glycolytic activity in chondrocytes exacerbates oxidative stress and impairs mitochondrial function, resulting in excessive ROS production in OA (osteoarthritis). Elevated ROS levels disrupt the cartilage ECM, trigger inflammatory responses, and ultimately drive OA progression [[17], [18], [19]].Collectively, these findings suggest that a shift toward enhanced oxidative phosphorylation in chondrocytes contributes to phenotypic instability by promoting excessive mitochondrial ROS generation.

Building upon the mechanisms discussed above, the development of an antioxidant-enriched regeneration system may offer a promising strategy to preserve the chondrocyte phenotype and enhance cartilage regeneration. By stabilizing chondrogenic metabolism, antioxidants can promote a glycolysis-dominant energy profile through the suppression of oxidative phosphorylation—a major source of ROS—thereby aligning with the intrinsic metabolic preferences of chondrocytes [20,21].

TA (Tannin acid), a naturally occurring polyphenol, has attracted increasing attention as a potent antioxidant due to its dual functionality in both scavenging ROS and inhibiting the mitochondrial ETC (electron transport chain), making it a promising candidate for managing oxidative stress-related disorders [22,23]. Previous reports demonstrated that TA-based hydrogels effectively promote the chondrogenic differentiation of bone marrow mesenchymal stem cells for articular cartilage repair [24]. However, the potential application of TA to maintain chondrocyte phenotype and facilitate cartilage regeneration through its antioxidant mechanisms has yet to be thoroughly investigated.

Following the selection of TA as the antioxidant agent for preserving the chondrocyte phenotype, the next critical step in this study was to develop an optimal biomaterial carrier for this small-molecule compound. In our previous work, we established a Col–PEG (dynamic collagen–polyethylene glycol) hydrogel system that demonstrated the ability to enhance chondrocyte migration and ECM deposition [25]. However, this system exhibited limitations, including suboptimal mechanical strength and a pronounced initial burst release of encapsulated agents.

To overcome these drawbacks, we developed a DN (double-net) hydrogel composed of Col–PEG and CNF (cellulose nanofibers), inspired by the double interpenetrating network architecture of proteoglycans in native articular cartilage. The primary network, formed by Col–PEG, provides dynamic properties that support cell proliferation and intercellular interactions. The secondary network, reinforced by CNF, features a nanofibrillar meshwork with outstanding mechanical properties and abundant surface hydroxyl groups that enable extensive hydrogen bonding with the collagen matrix. This structure significantly improves the mechanical strength and drug-loading capacity of the hydrogel. Finally, TA was incorporated into the hydrogel via hydrogen bonding and electrostatic interactions with CNF, resulting in the construction of an antioxidant-functionalized scaffold designed to support cartilage regeneration.

In summary, this study highlights the metabolic phenotype as a targetable axis in functional cartilage engineering and demonstrates that metabolic modulation represents a viable strategy for stabilizing chondrocyte phenotype and enhancing cartilage regeneration.

## 2 Results and discussion

### 2.1 *In vitro* expansion of chondrocytes induces aerobic metabolic shifts that contribute to phenotypic loss

Chondrocytes have a unique capacity to secrete cartilage-specific ECM and undergo spontaneous chondrogenesis, making them the most suitable seed cells for cartilage tissue engineering. However, these functional capacities are markedly diminished following *in vitro* expansion, resulting in suboptimal outcomes in regenerated cartilage constructs. Thus, preserving the chondrocyte phenotype during *in vitro* culture is a critical prerequisite for achieving clinically effective tissue-engineered cartilage.

In this study, we first investigated the mechanisms underlying chondrocyte phenotypic instability. To dissect the cellular and molecular changes occurring during expansion, we performed single-cell RNA sequencing on auricular chondrocytes at P1 (passage 1, representing well-maintained differentiated phenotypes) and P3 (passage 3, representing dedifferentiated phenotypes) with 3 biological replicates per group. Notably, in clinical practice, primary chondrocytes are typically expanded beyond 3 passages to obtain sufficient cell numbers for tissue engineering.

**Fig. 1:**
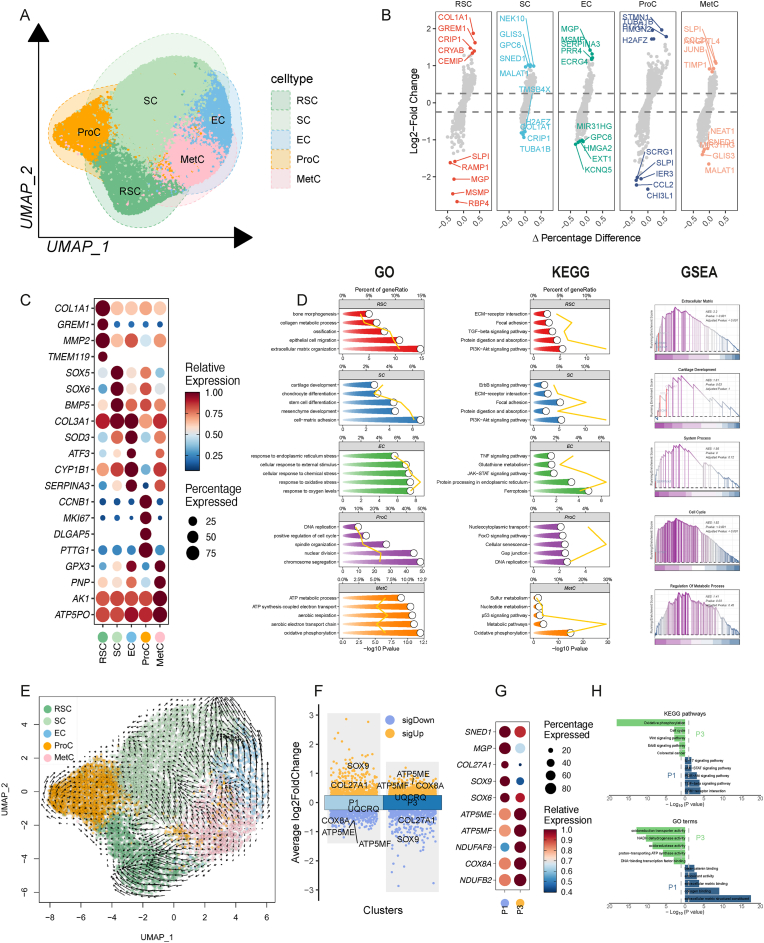
Identification of chondrocyte subtypes and dedifferentiated features based on single-cell RNA sequencing. (A) UMAP visualization of chondrocytes clustered into 5 distinct subtypes: RSCs, SCs, ECs, ProCs, and MetCs. (B) Volcano plot displaying DEGs among the 5 chondrocyte subtypes. Top 5 high and low expressed genes were marked. (C) Dot plot highlighting cell-type-specific marker genes for each chondrocyte subtype. (D) Functional enrichment analysis (GO, KEGG, and GSEA) of highly expressed genes, revealing unique biological functions associated with each cell type. (E) Cell differentiation trajectory analysis indicating that RSCs primarily originate from MetCs. (F) Volcano plot of DEGs between P1 and P3 cells. (G) Representative marker genes distinguishing P1 and P3 cells. Compared to P1, ECM-related genes (MGP, SNED1, SOX9, SOX6) were down-regulated. (H) KEGG and GO enrichment analysis of DEGs, illustrating important up-regulated oxidative phosphorylation metabolism level in P3. (UMAP: Uniform Manifold Approximation and Projection; RSC: Remodeling Stromal Cell; SC: Stromal Cell; EC: Effector Cells; ProC: Proliferative Cell; MetC: Metabolic Cell; DEG: Differentially expressed gene; GO: Gene Ontology; KEGG: Kyoto Encyclopedia of Genes and Genomes; GSEA: Gene Set Enrichment Analysis; P1: Passage 1; P3: Passage 3.)

Through comprehensive bioinformatic analysis, we identified five distinct chondrocyte subpopulations based on differentially expressed genes (Fig. 1A–C): RSCs (Remodeling Stromal Cells; *COL1A1, GREM1, MMP2, TMEM119*), SCs (Stromal Cells; *SOX5, SOX6, BMP5, COL3A1*), ECs (Effector Cells; *SOD3, ATF3, CYP1B1, SERPINA3*), ProCs (Proliferative Cells; *CCNB1, MKI67, DLGAP5, PTTG1*), and MetCs (Metabolic Cells; *GPX3, PNP, AK1, ATP5PO*). GO (Gene Ontology), KEGG (Kyoto Encyclopedia of Genes and Genomes), and GSEA (Gene Set Enrichment Analysis) revealed distinct biological functions associated with each subset (Fig. 1D). To validate the robustness of this subpopulation framework, we reanalyzed a publicly available murine articular chondrocyte scRNA-seq dataset, which yielded highly similar subpopulations with comparable distributions (Fig. S1).

Among these sub cell types, SCs serve as the primary contributors to chondrocyte-specific ECM synthesis, whereas RSCs are closely associated with phenotypic shifts. RSCs appear to mediate ECM remodeling from a cartilage-specific matrix to an ossifying or fibrotic ECM, thereby compromising chondrogenic potential, as evidenced by increased expression of *COL1A1, MMP2*, and *GREM1*(Fig. 1C). More importantly, pseudotime trajectory analysis revealed that RSCs predominantly originate from the MetC cluster (Fig. 1E), suggesting that metabolic reprogramming precedes phenotypic loss during expansion.

Comparative transcriptomic analysis between P1 and P3 chondrocytes further confirmed this hypothesis: P3 cells exhibited a marked upregulation of genes associated with aerobic metabolism (*ATP5PO, COX7A2L, NDUFA4*) alongside downregulation of genes involved in ECM synthesis (*MGP, SNED1, SOX9, SOX6*) (Fig. 1F–H), indicating that enhanced aerobic metabolism is closely linked to phenotypic deterioration.

Chondrocyte energy metabolic activity is tightly coupled to both ECM production and differentiation—key processes in cartilage regeneration. Previous studies have shown that reduced aerobic metabolic output can limit ATP production and impair cartilage repair, while excessive mitochondrial activity promotes ROS accumulation, leading to oxidative stress and cellular damage [[26], [27], [28]]. Ouyang et al. also demonstrated that enhanced aerobic metabolism exacerbates mitochondrial dysfunction and promotes dedifferentiation in articular chondrocytes [13]. Our data are consistent with these findings and further suggest that oxidative phosphorylation is a central driver of phenotypic instability in expanded chondrocytes. Notably, we also provide novel insights into the role of aerobic metabolism in ECM remodeling—a process previously underappreciated in chondrocyte de-differentiation studies.

In conclusion, these mechanistic insights underscore the importance of metabolic homeostasis in maintaining chondrocyte phenotype and lay a theoretical foundation for targeted metabolic interventions aimed at improving the clinical success of tissue-engineered cartilage.

### 2.2 Construction of double-network antioxidant hydrogel scaffolds

Given the dynamic metabolic requirements of chondrocytes at different physiological states [29,30], tailored metabolic modulation strategies are essential for maintaining their phenotypic stability. To attenuate excessive aerobic metabolism and preserve the chondrogenic phenotype, we sought to engineer an antioxidant-functionalized hydrogel scaffold.

TA, a naturally occurring polyphenol in the tannin family, has gained considerable attention due to its broad spectrum of biological activities, including anticancer, antioxidant, antimicrobial, and antiviral effects [22]. Previous studies have shown that tannins can effectively mitigate osteoarthritis progression [23] and delay chondrocyte dysfunction [31]. However, whether TA can modulate aerobic metabolism in chondrocytes both *in vitro* and *in vivo* remains unclear.

According to the STITCH database, in addition to scavenging ROS, TA can also bind to Nqo1, an enzyme responsible for catalyzing the reduction of quinones to reduce ROS generation, to further exert its antioxidant effects (Fig. S2A). This suggests that TA exerts its antioxidative effects via both chemical scavenging and enzymatic regulation pathways, offering a dual mechanism to counteract oxidative stress in chondrocytes.

Although preliminary evidence has confirmed the antioxidant efficacy of TA, the selection of an optimal delivery carrier to ensure its sustained release remains a critical challenge. Hydrogels, owing to their high water content and three-dimensional architecture that mimics native tissue, have been extensively utilized as classical scaffolds in cartilage regeneration. In this study, we employed a DN hydrogel system to encapsulate TA, thereby maximizing its antioxidant potential while providing mechanical support and cellular compatibility.

**Fig. 2:**
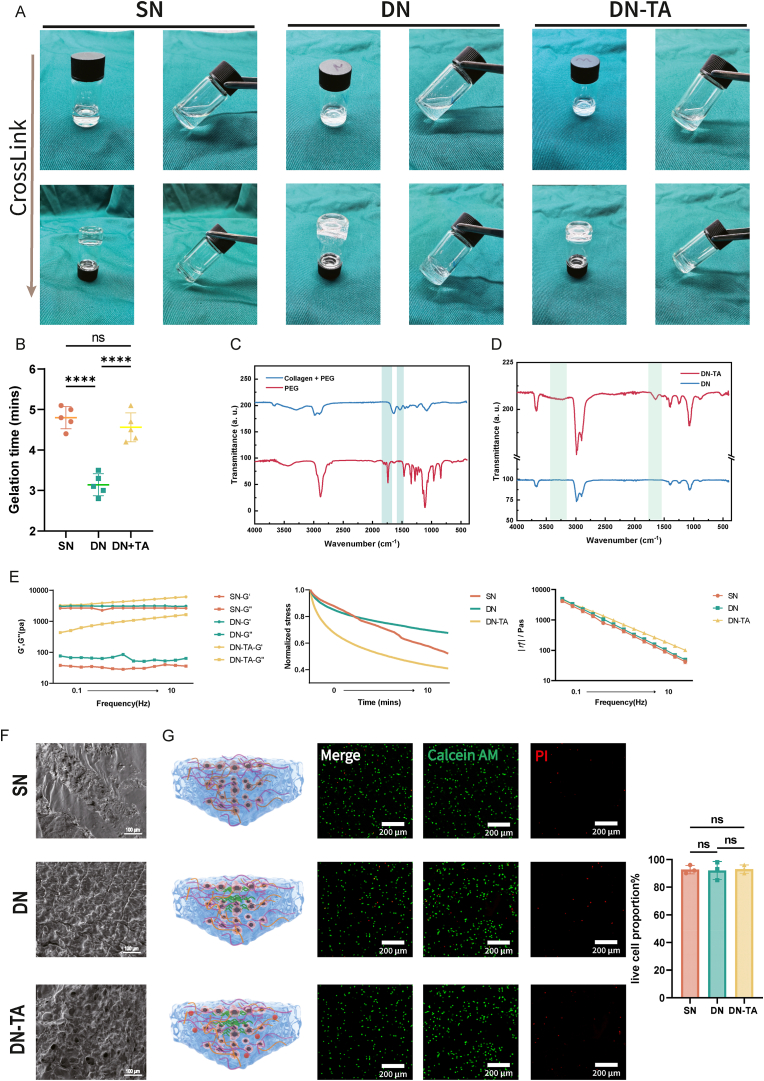
Construction and physicochemical properties of SN, DN, DN-TA hydrogels. (A) Crosslinking process of the three hydrogels. (B) Gelation time measurements(n = 5). (C) FTIR analysis of SN hydrogel, showing amide bond formation between collagen and PEG (C-O-C peak at 1100 cm-1; new peaks at 1650 cm-1 and 1550 cm-1). (D) FTIR analysis of DN-TA hydrogel, indicating hydrogen bonding (C=C stretching at 1600–1700 cm-1; broad O-H peak at 3200–3500 cm-1). (E) Rheological properties (G′, G″, stress relaxation, and frequency sweep curves) revealed up-regulated mechanical strength in DN and DN-TA hydrogels. (F) SEM images of hydrogel microstructures. (G) 3D culture of P3 cells in hydrogels, with Calcein AM/PI staining showing no significant difference in viability(n = 3). (SN: Single Net; DN: Double Net; TA: Tannin Acid; FTIR: Fourier Transform Infrared Spectroscopy; SEM: Scanning electron microscopy. Data are presented as means ± SD; Statistical analysis was performed using one-way ANOVA with Tukey's post hoc test as appropriate, ∗∗∗∗p < 0.0001, ns: no significance.)

The crosslinking schemes for all three hydrogel systems are illustrated in Fig. 2A and B. The SN (single-network) hydrogel was formed by covalently linking Collagen and PEG via amide bond formation (Fig. S2B), thereby establishing a biomimetic chondroid ECM microenvironment [[32], [33], [34]]. The successful conjugation of PEG to Collagen was confirmed by FTIR spectroscopy. Specifically, new absorbance peaks at 1650 cm^−1^ and 1550 cm^−1^ correspond to amide I and amide II bands, respectively, indicative of amide bond formation. Additionally, the retention of the characteristic C–O–C stretching peak at 1100 cm^−1^ further verified the presence of PEG in the conjugate (Fig. 2C).

This Col-PEG network exhibited favorable dynamic properties. Compared with traditional static hydrogels, dynamic hydrogels feature reversible crosslinking, allowing structural rearrangement under mechanical load. These properties facilitate stress relaxation and self-healing, thereby reducing mechanical stress on encapsulated cells. Our previous studies reveled that such dynamic behavior fosters a microenvironment conducive to cell viability, intercellular communication, and phenotypic stability—critical for preventing chondrocyte dedifferentiation [25].

To overcome the limitation of inadequate mechanical strength in dynamic hydrogels, a second network was introduced using CNF (Fig. S2C). DN hydrogel shower TA release rate compared to SN (Fig. S2D). CNF, a plant-derived, non-toxic, and biocompatible material, exhibits excellent mechanical strength, inherent antibacterial properties (Fig. S3A), and high surface area for drug adsorption. CNF was used to bind TA through hydrogen bonding, thus forming a robust double-network structure and improving TA retention. CNF has been widely adopted in biomedical applications such as wound dressings [35].

FTIR analysis confirmed the successful integration of TA into the DN-TA composite. Peaks between 1600 and 1700 cm^−1^ were attributed to the C=C stretching vibrations of TA's aromatic rings, indicating its presence. Moreover, the broadening of the O–H stretch at 3200–3500 cm^−1^ suggested the formation of hydrogen bonds between TA and the hydrogel matrix (Fig. 2D). Incorporation of both CNF and TA significantly enhanced the rheological properties of the scaffold. As shown in Fig. 2E, the storage modulus (G′) and loss modulus (G″) were markedly increased, indicating improved mechanical strength, elasticity, and viscosity of the DN-TA hydrogel.

SEM (Scanning electron microscopy) analysis further revealed distinct microstructural differences among the three hydrogel groups, highlighting the morphological impact of TA incorporation (Fig. 2F). The addition of TA resulted in a more compact and uniform porous structure, which is conducive to nutrient diffusion and cell–matrix interactions. These findings suggest that TA integration not only enhances the antioxidant properties of the hydrogel but also reinforces its mechanical and structural integrity.

Chondrocytes cultured within the SN, DN, and DN-TA hydrogels exhibited good biocompatibility, with no observable cytotoxicity or impairment in cell viability (Fig. 2G), thus validating the suitability of these scaffolds for further metabolic and functional analyses. Collectively, the double-network hydrogel system incorporating TA offers a promising platform that synergistically combines mechanical support with antioxidant functionality, potentially improving the efficacy of cartilage regeneration.

### 2.3 DN-TA hydrogel effectively reduces aerobic metabolism in P3 cells

**Fig. 3:**
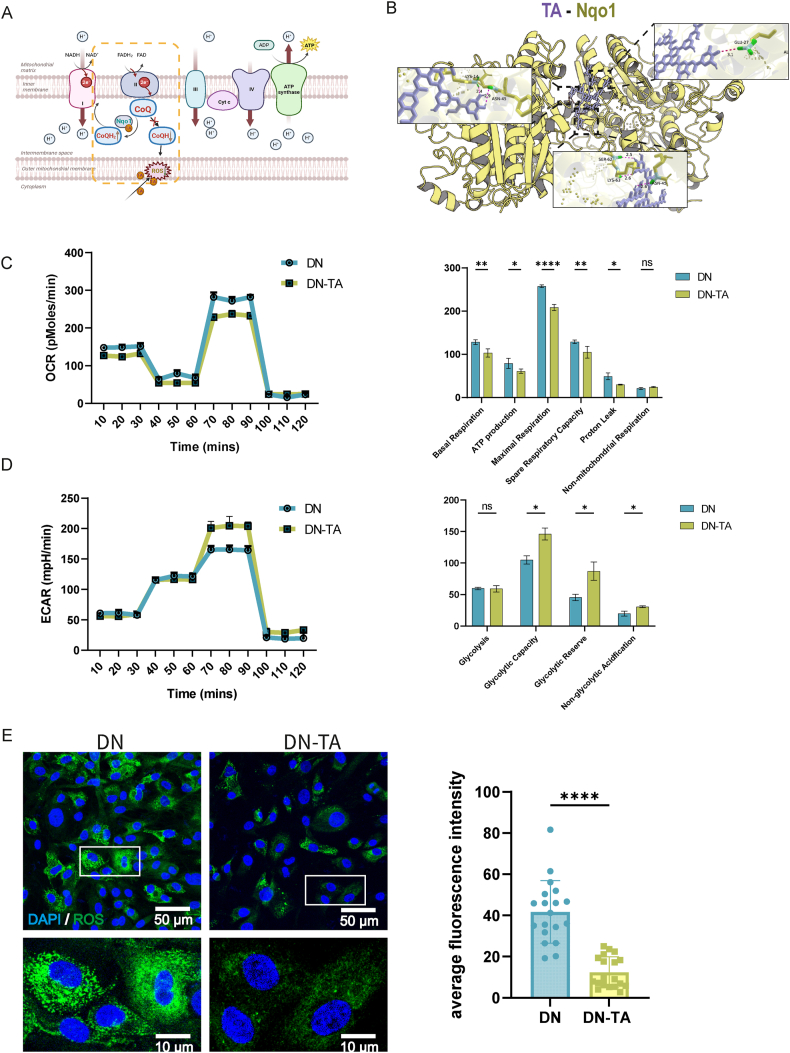
TA decreased aerobic metabolism level and ROS generation of P3 cells. (A) Schematic of TA's dual action: direct ROS scavenging and Nqo1-mediated ROS reduction. (B) Molecular auto-docking of tannin acid binding to Nqo1.(C) OCR curves and 6 key aerobic metabolism parameters for P3 cells in DN and DN-TA hydrogels. DN-TA exhibited significantly lower aerobic metabolism. (D) ECAR curves and 4 key parameters for P3 cells in DN and DN-TA hydrogels. (E)ROS staining of P3 cells in DN and DN-TA hydrogels. DN-TA demonstrated reduced ROS levels. (ROS: Reactive oxygen species; DN: Double Net; TA: Tannin acid; OCR: Oxygen Consumption Rate. ECAR: Extracellular Acidification Rate.Data are presented as means ± SD; Statistical analysis was performed using *t*-test, ∗p < 0.05, ∗∗p < 0.01, ∗∗∗p < 0.001, ∗∗∗∗p < 0.0001, ns: no significance.)

Following the successful fabrication of DN-TA hydrogels, we evaluated their regulatory effects on cellular metabolism using three-dimensional cultures of P3 cells. TA, as a potent antioxidant, can reduce ROS production via two distinct mechanisms. First, it directly scavenges ROS through chemical interaction [36,37]. Second, TA has been shown to interact with NQO1, facilitating a two-electron reduction of quinones to hydroquinones (CoQH2), thereby preventing the formation of semiquinone intermediates and suppressing mitochondrial ROS generation (Fig. 3A and B) [[38], [39], [40]].

Compared to DN group, the DN-TA hydrogel exhibited a pronounced suppressive effect on OCR (Oxygen Consumption Rate) curve. This was evidenced by significant reductions in basal respiration, ATP production, maximal respiration, spare respiratory capacity, and proton leak (Fig. 3C). In contrast, the glycolytic rate was elevated, as evidenced by an increase in the ECAR (Extracellular Acidification Rate, Fig. 3D). Furthermore, DN-TA hydrogels significantly scavenged intracellular ROS in P3 cells (Fig. 3E). These results collectively suggested that DN-TA hydrogels not only maintain high biocompatibility and support chondrocyte viability but also effectively attenuate excessive oxidative metabolism.

It is well established that chondrocytes predominantly rely on glycolysis for ATP generation, a metabolic adaptation essential for ECM synthesis and maintenance of the chondrogenic phenotype [[41], [42], [43]]. However, under *in vitro* culture conditions with elevated oxygen tension, chondrocytes undergo a metabolic shift toward oxidative phosphorylation. This metabolic reprogramming is often accompanied by phenotypic instability, characterized by reduced ECM production and impaired chondrogenic potential [44]. Moreover, increased oxidative metabolism has been implicated in cartilage degradation and the progression of osteoarthritis [45]. Taken together, these findings underscore the importance of restricting oxidative phosphorylation to preserve chondrocyte phenotype and promote successful cartilage regeneration.

### 2.4 DN-TA hydrogel enhances ECM deposition and upregulation of ECM-related genes

**Fig. 4:**
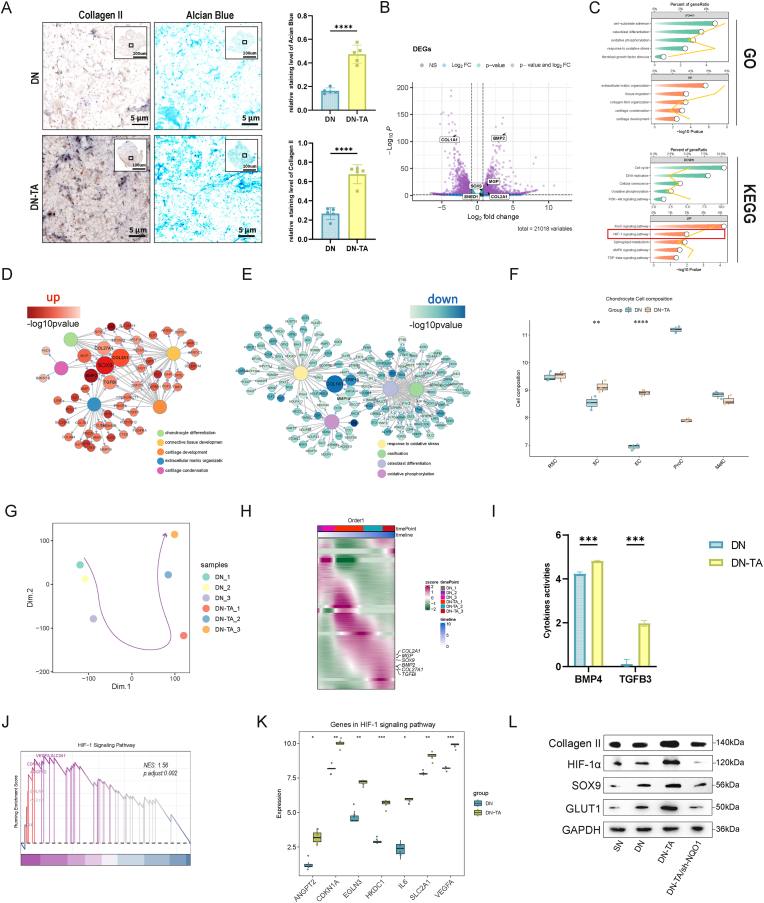
Effects of DN-TA hydrogel on ECM deposition and transcriptomic gene expression in P3 cells. (A) Alcian blue and collagen II staining of P3 cells in hydrogels. Quantitative analysis of staining intensity. TA significantly increased ECM deposition(n = 5). (B) Volcano plot of DEGs (DN-TA vs DN). Key ECM-related genes are highlighted. (C) GO/KEGG enrichment of DEGs. TA upregulated ECM genes while downregulating cell cycle and aerobic metabolism pathways. (D) Gene-BP network of upregulated genes, revealing central roles of SOX9, COL2A1 and BMP5. (E) Gene-BP network of downregulated genes, featuring COL1A1 and ATP5F1B as key nodes. (F) Bulk RNA-seq deconvolution identified significant SC cell-type enrichment in DN-TA group. (G) PCA trajectory analysis demonstrating distinct differentiation patterns. (H) Temporal gene expression heatmap. DN-TA group showed markedly elevated chondrogenic signature. (I) Cytokine activity prediction showed BMP4 and TGFB3 secretion was specifically enhanced in DN-TA group. (J) GSEA enrichment showed significant regulatory role of HIF-1 signaling pathway (K) Expression differences of genes in HIF-1 signaling pathway. (L) Changes induced by TA and NQO1 knock-down of protein expression related to HIF-1 pathway (HIF-1α, GLUT1) and cartilage phenotypes (Collagen II, SOX9). (ECM: Extracellular matrix; DN: Double Net; DEG: Differentially expressed genes; GO: Gene Ontology; KEGG: Kyoto Encyclopedia of Genes and Genomes; BP: Biological process; PCA: Principal component analysis; GSEA: Gene Set Enrichment Analysis; TA: Tannin Acid. Data are presented as means ± SD; Statistical analysis was performed using *t*-test, ∗p < 0.05, ∗∗p < 0.01, ∗∗∗p < 0.001, ∗∗∗∗p < 0.0001). (For interpretation of the references to colour in this figure legend, the reader is referred to the Web version of this article.)

To evaluate the effects of DN-TA hydrogel on cartilage regeneration, P3 cells were cultured on hydrogel scaffolds, and Alcian Blue and type II collagen staining were performed after 2 weeks (Fig. 4A). The results revealed markedly stronger staining in the DN-TA group compared to the DN group, indicating that the DN-TA scaffold significantly enhanced cartilage-specific ECM deposition.

To specificly elucidate the molecular mechanisms by which TA promotes ECM synthesis, we conducted bulk RNA-sequencing on DN and DN-TA groups. Transcriptomic analysis identified 1663 upregulated and 1439 downregulated genes (adjust.pvalue <0.05, log2foldchange >1) in the DN-TA group relative to the DN group (Fig. 4B, Fig. S3B). GO and KEGG enrichment analysis revealed that the upregulated genes were predominantly associated with chondrocyte differentiation and ECM production, whereas the downregulated genes were enriched in pathways related to oxidative metabolism, cellular proliferation, and ossification (Fig. 4C).

Gene-network analysis further demonstrated that DN-TA promoted ECM synthesis by upregulating central chondrogenic genes such as *COL2A1, SOX9*, and *BMP2*, while downregulating *COL1A1* and *ATP5F1B*, which are associated with fibrotic or osteogenic phenotypes and oxidative phosphorylation, respectively (Fig. 4D and E). Notably, Based on the marker genes identified from our previous chondrocyte subpopulation analysis, deconvolution analysis of the bulk RNA-seq data revealed that TA treatment significantly increased the proportion of ECM-related cell subpopulations (Fig. 4F). Pseudotime trajectory analysis confirmed a higher degree of chondrogenic differentiation in the DN-TA group, accompanied by progressive upregulation of *COL2A1, SOX9*, and *BMP2* along the differentiation timeline (Fig. 4G and H).

Additionally, cartilage-related cytokines including BMP4 and TGFβ3 were significantly elevated in the DN-TA group, further supporting TA's role in enhancing the chondrogenic microenvironment (Fig. 4I). Scissor analysis also indicated that TA increased SC-related cells(Fig. S3C). We speculate that the upregulation of cytokine expression (TGFβ3, BMPs) and expansion of SCs result from improved phenotypic stability of chondrocytes. This stabilization likely facilitates enhanced ECM production through autocrine and paracrine mechanisms.

To further elucidate the mechanism by which TA maintains the chondrocyte phenotype, we performed GSEA enrichment analysis and identified the HIF-1 signaling pathway as a potentially critical regulator in this process (Fig. 4J). As an oxygen-sensitive subunit, HIF-1α has been shown to regulate chondrocyte proliferation and differentiation, playing a pivotal role in preserving chondrocyte phenotype [[46], [47], [48]]. In the DN-TA group, we observed upregulation of multiple genes associated with the HIF-1 signaling pathway. Notably, SLC2A1, which encodes the glucose transporter GLUT1, plays a key role in supporting anaerobic glycolysis in chondrocytes (Fig. 4K). In addition, GLUT1 contributes significantly to extracellular matrix secretion and cartilage regeneration [18,49]. Our results confirmed that in the DN-TA group, HIF-1α, GLUT1, SOX9, and collagen II were all upregulated. However, upon NQO1 knockdown, these effects were reversed (Fig. 4L). Collectively, these findings suggest that TA enhances NQO1 activity, thereby activating the HIF-1 signaling pathway, which in turn upregulates GLUT1 expression. This metabolic reprogramming toward glycolysis promotes the maintenance of a healthy chondrocyte phenotype.

These findings indicate that DN-TA scaffolds effectively promote ECM synthesis and chondrogenic differentiation by modulating chondrocyte metabolism and phenotype maintenance.

### 2.5 Double-network antioxidant hydrogel scaffolds enhance cartilage regeneration

**Fig. 5:**
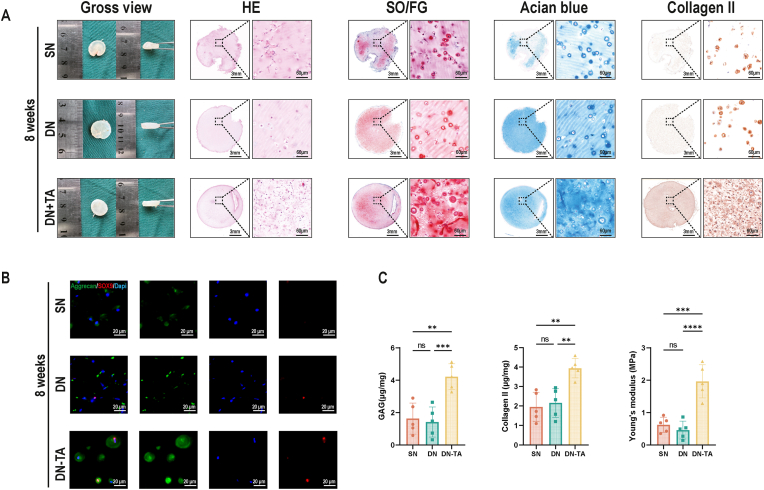
Cartilage regeneration for 8 weeks using DN and DN-TA hydrogels *in vivo*. (A) Macroscopic view and histological evaluation (HE, SO/FG, Collagen II) of regenerated cartilage 8 weeks post-implantation. DN-TA exhibits stronger ECM deposition. (B) Immunofluorescence images showed elevated aggrecan (green) and SOX9 (red) expression in DN-TA group. (C) Quantitative analysis of GAG/collagen II content and Young's modulus. (DN: Double Net; TA: Tannin acid; HE: Hematoxylin-eosin; SO/FG: Safranin O/Fast Green. ECM: Extracellular Matrix; GAG: Glycosaminoglycan. Data are presented as means ± SD; Statistical analysis was performed using one-way ANOVA (C) with Tukey's post hoc test as appropriate, ∗∗p < 0.01, ∗∗∗p < 0.001, ∗∗∗∗p < 0.0001). (For interpretation of the references to colour in this figure legend, the reader is referred to the Web version of this article.)

**Fig. 6:**
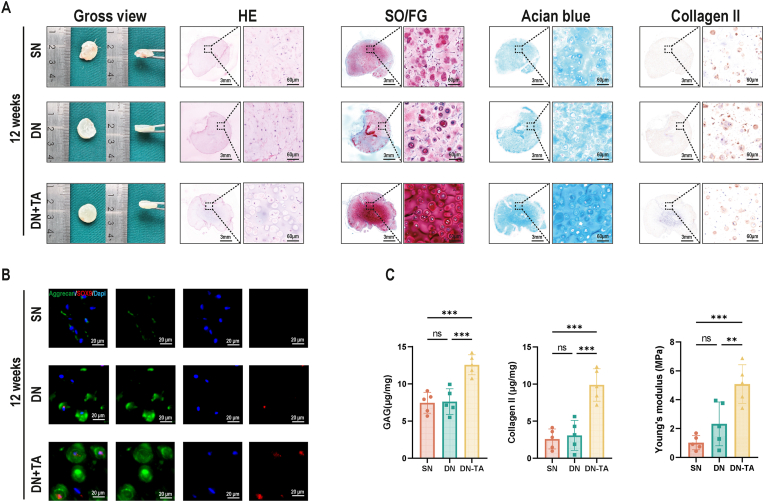
Cartilage regeneration for 12 weeks using DN and DN-TA hydrogels *in vivo*. (A) Macroscopic view and histological evaluation (HE, SO/FG, Collagen II) of regenerated cartilage 8 weeks post-implantation. DN-TA exhibits stronger ECM deposition. (B) Immunofluorescence images showed elevated aggrecan (green) and SOX9 (red) expression in DN-TA group. (C) Quantitative analysis of GAG/collagen II content and Young's modulus. (DN: Double Net; TA: Tannin acid; HE: Hematoxylin-eosin; SO/FG: Safranin O/Fast Green. ECM: Extracellular Matrix; GAG: Glycosaminoglycan. Data are presented as means ± SD; Statistical analysis was performed using one-way ANOVA (C) with Tukey's post hoc test as appropriate, ∗∗p < 0.01, ∗∗∗p < 0.001, ∗∗∗∗p < 0.0001). (For interpretation of the references to colour in this figure legend, the reader is referred to the Web version of this article.)

To verify whether the antioxidant DN-TA scaffold enhances cartilage regeneration, P3 chondrocytes were seeded into SN, DN, and DN-TA hydrogels and subcutaneously implanted into nude mice. At 8 (Fig. 5) and 12 (Fig. 6) weeks post-implantation, constructs were harvested for comprehensive evaluation of neocartilage formation.

Fig. 5, Fig. 6A show that all 3 groups developed mature cartilage-like tissue, characterized by an ivory-white appearance, visible lacunar structures, and abundant ECM deposition. Notably, the DN-TA group exhibited the most robust cartilage formation, with more defined lacunae and richer ECM, closely resembling native cartilage.To further assess chondrogenic capacity, we performed immunohistochemical staining for cartilage-specific markers. As shown in Fig. 5, Fig. 6B, the DN-TA group displayed significantly stronger staining for SOX9 and Aggrecan, indicating enhanced chondrogenic differentiation compared to the SN and DN groups.

Quantitative analysis of ECM components (GAG: glyeosaminoglycan and Collagen II) confirmed these findings. The DN-TA group demonstrated significantly greater ECM deposition than the SN and DN groups. In addition, mechanical testing revealed that the regenerated tissue in the DN-TA group exhibited superior biomechanical properties, further supporting its enhanced cartilage regeneration potential (Fig. 5, Fig. 6C).

Together, these results indicate that the incorporation of antioxidant tannic acid into the double-network hydrogel scaffold effectively maintains chondrocyte phenotype, enhances ECM production, and improves mechanical integrity of the regenerated cartilage.

Moreover, while conventional DN group offered modest improvements over the SN group, only the DN-TA group achieved significant enhancements in both biological and mechanical outcomes. These findings underscore the critical role of metabolic modulation in tissue engineering.

As an emerging strategy, double-network hydrogels provide tunable mechanics and bioactivity through synergistic network design [[50], [51], [52]]. Building on our previous work [25], this study demonstrates that incorporating antioxidant functionality into DN hydrogels confers significant benefits. Unlike conventional DN systems with limited phenotype-stabilizing effects, the DN-TA scaffold delivers sustained chondroprotective outcomes by reducing aerobic metabolism and stabilizing chondrogenic activity.

The DN-TA hydrogel scaffold exhibits considerable clinical translational potential owing to its excellent biocompatibility, tunable mechanical properties, and sustained antioxidant activity. Its demonstrated ability to preserve the chondrocyte phenotype and promote cartilage regeneration suggests broad applicability in reconstructive procedures, particularly for maintaining complex anatomical structures such as the auricle. Moreover, its antioxidant properties may offer therapeutic benefits in the treatment of bone and joint disorders by facilitating defect filling and tissue regeneration. Nevertheless, these promising findings require further validation in large animal models to support future clinical application.

In summary, this study confirms that excessive aerobic metabolism undermines chondrocyte phenotype and presents antioxidant-functionalized DN hydrogels as a promising platform for engineering functional cartilage.

Although this study has demonstrated the effectiveness of DN-TA hydrogels in promoting cartilage regeneration, significant challenges remain before clinical translation can be fully realized. The regenerative capacity of DN-TA scaffolds for anatomically complex cartilage structures—such as the auricle—or their therapeutic potential in repairing articular cartilage defects has yet to be thoroughly investigated. Future studies will focus on evaluating the performance of DN-TA scaffolds in large animal models, including the regeneration of complex-shaped cartilage and the repair of joint cartilage defects, to further assess their clinical applicability.

## 3 Conclusion

In this study, single-cell RNA sequencing of *in vitro* expanded chondrocytes revealed that excessive aerobic metabolism is a critical driver of chondrocyte dedifferentiation, thereby compromising their regenerative potential. To overcome this challenge, we engineered a DN-TA hydrogel scaffold, which effectively attenuates oxidative metabolism and enhances cartilage regeneration. These findings highlight the importance of designing biomaterials tailored to the phenotypic and metabolic states of cells, offering a promising strategy for improving the efficacy of tissue-engineered cartilage and facilitating clinical translation.

## 4 Materials and methods

### 4.1 Isolation, culture, expansion and cryopreservation of auricular chondrocytes

Cartilage tissues were obtained from residual ear cartilage samples collected from patients undergoing tympanoplasty at the Eye and ENT Hospital of Fudan University. The isolation, culture, expansion, and cryopreservation of chondrocytes were carried out following previously published protocols [5,8]. Cartilage tissues were dissected to remove fibrous tissue, minced, and digested with 0.15 % collagenase (Sigma-Aldrich, USA) to isolate chondrocytes. Cells were seeded at 1 × 10^6^ cells per 10 cm dish in high-glucose DMEM (HyClone) containing 10 % FBS, and passaged at a 1:3 ratio.

### 4.2 Single cell sequencing

P1 and P3 Chondrocytes were enzymatically digested into a single-cell suspension and combined with 10 × barcodes and Master Mix (10 × Genomics Chromium, Capital Biotechnology, Beijing, China). The single-cell library was prepared following the manufacturer's protocol, and high-throughput sequencing was conducted on the Illumina NovaSeq platform.

### 4.3 Single cell transcriptome data quantifications and analysis

Raw reads were processed to generate gene expression profiles, with cell barcodes and UMIs extracted after filtering. Read2 sequences were mapped to the GRCh38 genome using Cell Ranger with STAR (V2.7.11), and UMIs were counted for each gene per cell. Seurat (V4.2.0) [16] was used for data analysis, including normalization, PCA, and batch correction using Harmony. Functions such as "FindNeighbors," "FindClusters," and "runUMAP" visualized cell types, while "FindMarker" identified differentially expressed genes. GO, KEGG and GSEA enrichment analyses were performed using the R package clusterProfiler (V4.9.1). Cell differentiation trajectory were measured by software scVelo (V0.3.2).

### 4.4 Construction of SN, DN and DN-TA hydrogel

SN hydrogel was constructed by PEG and collagen following our previous research [32]. For DN and DN-TA construction, a hydrogel precursor solution (Solution A) was prepared by dissolving collagen and nanofibers (NF) in phosphate buffered saline (PBS) at pH 7.2–7.4 to the desired concentrations. Separately, another precursor solution (Solution B) was prepared by dissolving 4-arm-PEG-SC (with or without TA) in PBS at pH 7.2–7.4. Equal volumes of Solution A and Solution B were then mixed and vortexed thoroughly. Prior to gelation, the precursor solution could be injected into various molds or cell culture plates. For cell culture or animal experiments, the solution was sterilized by filtration through a 0.2 μm syringe filter in a biosafety cabinet.

### 4.5 Rotational rheometry

The rheological behavior of the hydrogel was characterized using a rotational rheometer (MARS III Haake). The hydrogel precursor solution was poured into a mold to form a disk with a diameter of 20 mm and a height of 1 mm. The rheological measurements were conducted at 37 °C with a 1 mm gap between the cone and plate. An amplitude sweep test was first performed to determine the LVR (linear viscoelastic region), where the modulus of the hydrogel is independent of the strain amplitude. Subsequently, a frequency sweep test was conducted at a fixed strain amplitude within the LVR, covering a frequency range of 0.1–10 Hz. The storage modulus (G′) and loss modulus (G″) of the hydrogel were obtained (n = 5).

### 4.6 TA release assay

SN and DN hydrogels (100 mg, containing 238 μg and 119 μg TA, respectively) were incubated in 20 mL of PBS (pH 7.4) at 37 °C with shaking (100 rpm). At predetermined time points, 2 mL of the medium was collected for UV–vis analysis at 278 nm and replaced with fresh PBS. TA concentration was determined using a standard calibration curve, and cumulative release was calculated. All experiments were conducted in triplicate.

### 4.7 FTIR (Fourier Transform Infrared Spectroscopy) and SEM (scanning electron microscopy)

The hydrogel was freeze-dried, ground with KBr, and pressed into a pellet for FTIR analysis. The FTIR spectra were collected in the range of 4000-400 cm^−1^ to identify the chemical components of the hydrogel.

To observe the microporous structure of the hydrogel, the freeze-dried samples were further frozen in liquid nitrogen and fractured. The fractured surfaces were then mounted on a SEM stub and coated with a thin layer of gold. The morphology of the hydrogel surface and cross-section was observed using an SEM (S-3400) at an accelerating voltage of 10 kV.

### 4.8 3D culture using hydrogel of chondrocytes and cell viability, aerobic metabolism level and ROS measurement

The P3 cells were mixed with hydrogel at a concentration of 10^7^/mL before crosslinking and then crosslinked.

For Calcein AM, dilute the stock solution to approximately 2 μM in culture medium, and for PI, dilute to a final concentration of around 5 μg/mL(Beyotime, China). Add the staining solution to the cells and incubate for 15–30 min at 37 °C in the dark, followed by gentle washing with PBS to remove excess dye (n = 3).

For ROS probe, prepare the ROS probe solution (Beyotime, China) according to the manufacturer's instructions, add it to the cells, and incubate for 30 min at 37 °C, followed by another wash with PBS. After staining, visualize the cells using a fluorescence or confocal microscope. Finally, analyze the staining patterns to assess cell viability and oxidative stress(n = 3). The OCR of chondrocytes were measured in Seahorse extracellular flux analyzer (XFe24, Agilent Technologies). Initially, cells were cultured and allowed to adhere to the surface. Subsequently, media was refreshed and specific concentrations of respiratory chain inhibitors were added into subsequent assays.

For OCR, 1 μmol·L−1 oligomycin, 1 μmol·L−1 FCCP and 1 μmol·L−1 antimycin were added. 3 replicates for each condition were used. For ECAR, 100 mmol·L−1 glucose, 100 μmol·L−1 oligomycin and 500 mmol·L−1 2-2-deoxyglucose (2-DG) for ECAR. All testing process were followed by standard protocols (https://www.agilent.com).

### 4.9 Bulk-RNA sequencing and data analysis

After culturing in DN and DN-TA hydrogel for 2 weeks, total RNA were obtained for bulk RNA sequencing. RNA was washed with 70 % ethanol, air-dried, and dissolved in RNase-free water. Quality and quantity were assessed by NanoDrop. For each group, 1.5 μg of RNA was used for sequencing on the Illumina NovaSeq 6000 platform. After quality control with FastQC, reads were aligned using HISAT2, and transcript abundance was estimated with StringTie. DEGs were identified using the DESeq2 package. Pseudo-time for all samples were measured following previous published articles [32,53].

### 4.10 Immunoblotting and NQO1 knock-down

Chondrocytes were lysed in RIPA buffer with protease inhibitors. Proteins were separated by SDS-PAGE, transferred to PVDF membranes, and blocked. Membranes were incubated overnight at 4 °C with primary antibodies, followed by HRP-conjugated secondary antibodies. Bands were detected by enhanced chemiluminescence and imaged using GeneSys software.

NQO1 knockdown was performed using a final concentration of 50 μm mol·L−1 shRNA (Hanbio, Shanghai, China).

### 4.11 Cartilage regeneration *in vivo*, immunofluorescence and immunohistochemical staining

Male nude mice (4–6 weeks old, Jiangsu GemPharmatech) were housed under standard conditions with free access to food and water. All procedures were approved by the Ethics Committee of the Eye & ENT Hospital of Fudan University (No. 2017043) and complied with NIH animal care guidelines. Sample size was calculated using G∗Power (effect size d = 2.0, α = 0.05, power = 0.8), requiring n = 5 per group.

Mice were randomly assigned into 3 groups. For *in vivo* cartilage regeneration, P3 cells were encapsulated in SN, DN and DN-TA hydrogels (1 × 10^7^ cells/mL), then subcutaneously injected into the dorsal region. After 8 or 12 weeks, regenerated tissues were collected for gross and histological analysis. Group allocation was known to experimenters, but outcome assessment was blinded. Initial injections were done without anesthesia; tissue collection used isoflurane anesthesia.

Regenerated cartilage tissues collected from nude mice were fixed, embedded in paraffin, and sectioned at 5 μm thickness.For immunofluorescence, samples were fixed on glass slides were washed with PBS, incubated overnight at 4 °C with primary antibodies (Aggrecan and SOX9, Preoteintech, USA), then with Alexa Fluor 488/555-conjugated secondary antibodies for 1 h at room temperature in the dark. Nuclei were stained with DAPI, and images were captured using a confocal laser scanning microscope (Zeiss, USA).

For immunohistochemistry, sections were stained with hematoxylin and eosin (H&E), Safranin O/Fast Green (SO/FG), Alcian blue and immunostained for collagen II (Proteintech, USA).

### 4.12 GAG and collagen II content quantitative analysis

Briefly, GAG content was quantified using the alcian blue assay (GENMED SCIENTIFICS, USA) and Protein levels of collagen II were determined using ELISA kits according to the manufacturer's instructions (CUSABIO, Wuhan, China).

### 4.13 Statistical analysis

All the data were presented as the means ± SD. Normality tests were conducted to confirm the normal distribution of data for parametric statistical analysis. Statistical differences were assessed using one way ANOVA or t-tests. All statistical analyses were performed with GraphPad Prism 10 (GraphPad Software, USA). A p-value of less than 0.05 was considered statistically significant.

## CRediT authorship contribution statement

**Xu Wu:** Writing – original draft, Software, Resources, Methodology, Investigation, Formal analysis, Conceptualization. **Honglei Wang:** Writing – review & editing, Writing – original draft, Visualization, Validation, Methodology, Investigation, Conceptualization. **Chenlong Li:** Supervision, Resources. **Yaying Zhu:** Writing – review & editing, Resources, Methodology. **Qixuan Wang:** Visualization, Formal analysis. **Tianyu Zhang:** Writing – review & editing, Supervision, Project administration, Funding acquisition, Conceptualization. **Yaoyao Fu:** Writing – review & editing, Supervision, Funding acquisition, Conceptualization. **Aijuan He:** Writing – review & editing, Supervision, Funding acquisition, Conceptualization.

## Ethics approval

Ethical approval for the study was obtained from the medical ethics committee of the Eye & ENT Hospital of Fudan University (approval number: 2017043). All participants provided written informed consent prior to their inclusion in the study. The animal studies were performed after receiving approval of Institutional Animal Care of Eye & ENT Hospital of Fudan University.

## Funding

This work was supported by 10.13039/501100001809National Natural Science Foundation of China (82172105), Natural Science Foundation of Shanghai (21DZ2200700, 20ZR1409900), and Double Excellent Foundation of Eye & ENT Hospital (SYA202003).

## Declaration of competing interest

All authors declared no conflict interests.

## Data Availability

Single-cell RNA-seq data was uploaded to GEO database (GSE270642). Bulk RNA-seq data was uploaded to GEO database (GSE270646).

## Abbreviations

DN: Double Net
SN: Single Net
UMAP: Uniform Manifold Approximation and Projection
RSC: Remodeling Stromal Cell
SC: Stromal Cell
EC: Effector Cells
ProC: Proliferative Cell
MetC: Metabolic Cell
DEG: Differentially expressed gene
GO: Gene Ontology
KEGG: Kyoto Encyclopedia of Genes and Genomes
GSEA: Gene Set Enrichment Analysis
P1: Passage1
P3: Passage 3
TA: Tannin Acid
FTIR: Fourier Transform Infrared Spectroscopy
SEM: Scanning electron microscopy
ROS: Reactive Oxygen Species
ECM: Extracellular matrix
BP: Biological process
PCA: Principal component analysis
HE: Hematoxylin-eosin
SO/FG: Safranin O/Fast Green
GAG: Glycosaminoglycan
LVR: linear viscoelastic region

## References

[1] Sterodimas A., de Faria J. (2013). Human auricular tissue engineering in an immunocompetent animal model. Aesthetic Surg. J..

[2] Zhou G., Jiang H., Yin Z., Liu Y., Zhang Q., Zhang C., Pan B., Zhou J., Zhou X., Sun H., Li D., He A., Zhang Z., Zhang W., Liu W., Cao Y. (2018). In vitro regeneration of patient-specific ear-shaped cartilage and its first clinical application for auricular reconstruction. EBioMedicine.

[3] Cao Y., Vacanti J.P., Paige K.T., Upton J., Vacanti C.A. (1997). Transplantation of chondrocytes utilizing a polymer-cell construct to produce tissue-engineered cartilage in the shape of a human ear. Plast. Reconstr. Surg..

[4] Jia L.T., Hua Y.J., Zeng J.S., Liu W.S., Wang D., Zhou G.D., Liu X., Jiang H.Y. (2022). Bioprinting and regeneration of auricular cartilage using a bioactive bioink based on microporous photocrosslinkable acellular cartilage matrix. Bioact. Mater..

[5] He A.J., Ye A.Q., Song N., Liu N.H., Zhou G.D., Liu Y.Q., Ye X.H. (2020). Phenotypic redifferentiation of dedifferentiated microtia chondrocytes through a three-dimensional chondrogenic culture system. Am J Transl Res.

[6] C H., Y P., L J., S Y., L H., L P., W H., L A., G B., T Y., B X., L K. (2023). MYL3 protects chondrocytes from senescence by inhibiting clathrin-mediated endocytosis and activating of notch signaling. J.N. communications.

[7] Ma B., Leijten J.C.H., Wu L., Kip M., van Blitterswijk C.A., Post J.N., Karperien M. (2013). Gene expression profiling of dedifferentiated human articular chondrocytes in monolayer culture. Osteoarthr. Cartil..

[8] Wu X., Fu Y., Ma J., Li C., He A., Zhang T. (2024). LGR5 modulates differentiated phenotypes of chondrocytes through PI3K/AKT signaling pathway. Tissue Eng Regen Med null.

[9] W X., F Y., M J., W H., L C., Z Y., W Q., G X., Z T., H A. (2025). Blocking calcium-MYC regulatory axis inhibits early dedifferentiation of chondrocytes and contributes to cartilage regeneration. Stem Cell Res. Ther..

[10] Otto I.A., Bernal P.N., Rikkers M., van Rijen M.H.P., Mensinga A., Kon M., Breugem C.C., Levato R., Malda J. (2022). Human adult, pediatric and microtia auricular cartilage harbor fibronectin-adhering progenitor cells with regenerative ear reconstruction potential. iScience.

[11] Wuest S.L., Calio M., Wernas T., Tanner S., Giger-Lange C., Wyss F., Ille F., Gantenbein B., Egli M. (2018). Influence of mechanical unloading on articular chondrocyte dedifferentiation. Int. J. Mol. Sci..

[12] Meng F., Li Z., Zhang Z., Yang Z., Kang Y., Zhao X., Long D., Hu S., Gu M., He S., Wu P., Chang Z., He A., Liao W. (2018). MicroRNA-193b-3p regulates chondrogenesis and chondrocyte metabolism by targeting HDAC3. Theranostics.

[13] Chen Y.S., Yu Y.K., Wen Y., Chen J., Lin J.X., Sheng Z.X., Zhou W.Y., Sun H., An C.R., Chen J.S., Wu W.L., Teng C., Wei W., Ouyang H.W. (2022). A high-resolution route map reveals distinct stages of chondrocyte dedifferentiation for cartilage regeneration. Bone Res.

[14] Zheng L., Zhang Z., Sheng P., Mobasheri A. (2021). The role of metabolism in chondrocyte dysfunction and the progression of osteoarthritis. Ageing Res. Rev..

[15] Zhang F., Zhang B., Wang Y., Jiang R., Liu J., Wei Y., Gao X., Zhu Y., Wang X., Sun M., Kang J., Liu Y., You G., Wei D., Xin J., Bao J., Wang M., Gu Y., Wang Z., Ye J., Guo S. (2023). An extra-erythrocyte role of haemoglobin body in chondrocyte hypoxia adaption. Nature.

[16] Xue C., Tian J., Cui Z., Liu Y., Sun D., Xiong M., Yi N., Wang K., Li X., Wang Y., Xu H., Zhang W., Liang Q. (2024). Reactive oxygen species (ROS)-mediated M1 macrophage-dependent nanomedicine remodels inflammatory microenvironment for osteoarthritis recession. Bioact. Mater..

[17] He X.X., Huang Y.J., Hu C.L., Xu Q.Q., Wei Q.J. (2024). Songorine modulates macrophage polarization and metabolic reprogramming to alleviate inflammation in osteoarthritis. Front. Immunol..

[18] Li K., Ji X., Seeley R., Lee W.C., Shi Y., Song F., Liao X., Song C., Huang X., Rux D., Cao J., Luo X., Anderson S.M., Huang W., Long F. (2022). Impaired glucose metabolism underlies articular cartilage degeneration in osteoarthritis. FASEB J..

[19] Zhang J., Gao P., Chang W.R., Song J.Y., An F.Y., Wang Y.J., Xiao Z.P., Jin H., Zhang X.H., Yan C.L. (2025). The role of HIF-1α in hypoxic metabolic reprogramming in osteoarthritis. Pharmacol. Res..

[20] Zhang S., Wang L., Kang Y., Wu J., Zhang Z. (2023). Nanomaterial-based reactive oxygen species scavengers for osteoarthritis therapy. Acta Biomater..

[21] Jiang Z., Wang H., Zhang Z., Pan J., Yuan H. (2022). Cartilage targeting therapy with reactive oxygen species-responsive nanocarrier for osteoarthritis. J. Nanobiotechnol..

[22] Guan X., Zhang B., Wang Z., Han Q., An M., Ueda M., Ito Y. (2023). Natural polyphenol tannin-immobilized composites: rational design and versatile applications. J. Mater. Chem. B.

[23] Govoni M., Danesi F. (2022). Do pomegranate hydrolyzable tannins and their derived metabolites provide relief in osteoarthritis? Findings from a scoping review. Molecules.

[24] Li Y., Chen M., Yan J., Zhou W., Gao S., Liu S., Li Q., Zheng Y., Cheng Y., Guo Q. (2021). Tannic acid/Sr 2+ -coated silk/graphene oxide-based meniscus scaffold with anti-inflammatory and anti-ROS functions for cartilage protection and delaying osteoarthritis. Acta Biomater..

[25] Wang H., Wu X., Chen L., Tong H., Hu X., He A., Li C., Guo X., Fu Y., Zhang T. (2025). Dynamic Col-HZ hydrogel with efficient delivery of bioactivator promotes ECM deposition and cartilage formation. Mater. Today Bio.

[26] Court A.C., Vega-Letter A.M., Parra-Crisóstomo E., Velarde F., García C., Ortloff A., Vernal R., Pradenas C., Luz-Crawford P., Khoury M., Figueroa F.E. (2024). Mitochondrial transfer balances cell redox, energy and metabolic homeostasis in the osteoarthritic chondrocyte preserving cartilage integrity. Theranostics.

[27] Wu X., Liyanage C., Plan M., Stark T., McCubbin T., Barrero R.A., Batra J., Crawford R., Xiao Y., Prasadam I. (2023). Dysregulated energy metabolism impairs chondrocyte function in osteoarthritis. Osteoarthr. Cartil..

[28] Fermor B., Gurumurthy A., Diekman B.O. (2010). Hypoxia, RONS and energy metabolism in articular cartilage. Osteoarthr. Cartil..

[29] Sakata S., Kunimatsu R., Tanimoto K. (2024). Protective effect of ergothioneine against oxidative stress-induced chondrocyte death. Antioxidants.

[30] Xin R., Xu Y., Long D., Mao G., Liao H., Zhang Z., Kang Y. (2022). Mitochonic Acid-5 inhibits reactive oxygen species production and improves human chondrocyte survival by upregulating SIRT3-Mediated, parkin-dependent mitophagy. Front. Pharmacol..

[31] Yang L., Fan C., Shu T., Wang S. (2021). Punicalin alleviates TNF-α- and IL-1β-induced chondrocyte dysfunction and cartilage metabolism via mediating FOXO3 signaling axis. J Food Biochem null.

[32] Honglei W., Runzhi H., Long B., Yong C., Lei M., Chunyan B., Shaoliang L., Shizhao J., Changsheng L., Xue Q. (2023). Extracellular matrix‐mimetic immunomodulatory hydrogel for accelerating wound healing. Adv. Healthcare Mater..

[33] Lee H., Hong H.J., Ahn S., Kim D., Kang S.H., Cho K., Koh W.G. (2023). One-pot synthesis of double-network PEG/collagen hydrogel for enhanced adipogenic differentiation and retrieval of adipose-derived stem cells. Polymers.

[34] Fernandes-Cunha G.M., Chen K.M., Chen F., Le P., Han J.H., Mahajan L.A., Lee H.J., Na K.S., Myung D. (2020). In situ-forming collagen hydrogel crosslinked via multi-functional PEG as a matrix therapy for corneal defects. Sci. Rep..

[35] Chen R., Zhao C., Chen Z., Shi X., Zhu H., Bu Q., Wang L., Wang C., He H. (2022). A bionic cellulose nanofiber-based nanocage wound dressing for NIR-triggered multiple synergistic therapy of tumors and infected wounds. Biomaterials.

[36] Allie R., Savita C. (2022). Antioxidant activity and Reactive Oxygen Species (ROS) scavenging mechanism of eriodictyon californium, an edible herb of North America. J. Chem..

[37] Khaled M.A.R., Hossam S.E.-B., Heba I.M., Tarek S., Galal A., Abdallah Tageldein M., Mohamed M.A.F., Eslam S.A.B. (2022). Antioxidant, anti-cancer activity and phytochemicals profiling of Kigelia pinnata fruits. Separations.

[38] Serdar K., Melike S., Hasan Ufuk Ç., Orhan A. (2015). Alteration of enzyme activities and kinetic properties of GST and NQO1 with naturally occurring phenolic compounds. Türk Biyokim. Derg..

[39] Ting L., Tedd M.G., Neil F.S. (2020). Metabolic syndrome is reduced in C57BL/6J mice fed high-fat diets supplemented with oak tannins. Curr. Dev. Nutr..

[40] Peng Y., Xiaojie R., Jiaxing N., Yang L., Libo H., Shuzhen J., Ning J., Xuejun Y., Weiren Y., Yang L. (2023). Effects of dietary Galla chinensis tannin supplementation on antioxidant capacity and intestinal microbiota composition in broilers. Agriculture.

[41] Kai Z., Fengjie Z., Wing Pui T., Wai Yee C., Chao W. (2016). Hypoxia enhances engineered chondrogenesis through coordinating chondrocyte glucose metabolism and differentiation. Journal of Orthopaedic Translation.

[42] Li M., Ning J., Wang J., Yan Q., Zhao K., Jia X. (2021). SETD7 regulates chondrocyte differentiation and glycolysis via the hippo signaling pathway and HIF-1α. Int. J. Mol. Med..

[43] Wang P., Xiong X., Zhang J., Qin S., Wang W., Liu Z. (2020). Icariin increases chondrocyte vitality by promoting hypoxia-inducible factor-1α expression and anaerobic glycolysis. Knee.

[44] Bo‐Hao C., Qi H., Chuyi C., Yuewei L., Jiacong X., Zhaofeng P., Miao L., Shaocong L., Junzheng Y., FanChen W., Jiaxu Z., Yanzi Y., Weijin C., Ke M., Haibin W., Peng C. (2023). Combination of curcumin and catalase protects against chondrocyte injury and knee osteoarthritis progression by suppressing oxidative stress. Biomed. Pharmacother..

[45] Liao C.R., Wang S.N., Zhu S.Y., Wang Y.Q., Li Z.Z., Liu Z.Y., Jiang W.S., Chen J.T., Wu Q. (2020). Advanced oxidation protein products increase TNF-α and IL-1β expression in chondrocytes via NADPH oxidase 4 and accelerate cartilage degeneration in osteoarthritis progression. Redox Biol..

[46] Schipani E., Ryan H.E., Didrickson S., Kobayashi T., Knight M., Johnson R.S. (2001). Hypoxia in cartilage: HIF-1alpha is essential for chondrocyte growth arrest and survival. Genes Dev..

[47] Z J., J L., M S., Y L., N S., K X., X L., D Z., X L., Z Z., H D. (2025). Hypoxia, cuproptosis, and osteoarthritis: unraveling the molecular crosstalk. Redox Biol..

[48] Li S., Luo D., Liang Y., Zou Y., Pu H., Zheng M., Wang Y., Sun X., Zhu H., Zhu Y., Zhao L., Xiao J. (2025). BCLAF1 regulates osteoarthritic cartilage degradation through interaction with LAMTOR2. Int. J. Biol. Sci..

[49] Mobasheri A., Richardson S., Mobasheri R., Shakibaei M., Hoyland J.A. (2005). Hypoxia inducible factor-1 and facilitative glucose transporters GLUT1 and GLUT3: putative molecular components of the oxygen and glucose sensing apparatus in articular chondrocytes. Histol. Histopathol..

[50] Li G., Huang K., Deng J., Guo M., Cai M., Zhang Y., Guo C.F. (2022). Highly conducting and stretchable double-network hydrogel for soft bioelectronics. Adv. Mater..

[51] Bauman L., Zhao B. (2023). Multi-thermo responsive double network composite hydrogel for 3D printing medical hydrogel mask. J. Colloid Interface Sci..

[52] Chen Y.R., Yan X., Yuan F.Z., Lin L., Wang S.J., Ye J., Zhang J.Y., Yang M., Wu D.C., Wang X., Yu J.K. (2022). Kartogenin-conjugated double-network hydrogel combined with stem cell transplantation and tracing for cartilage repair. Adv. Sci. (Weinh.).

[53] Liu X., Bie X.M., Lin X., Li M., Wang H., Zhang X., Yang Y., Zhang C., Zhang X.S., Xiao J. (2023). Uncovering the transcriptional regulatory network involved in boosting wheat regeneration and transformation. Nat. Plants.

