# Supplementary material for: Antioxidant functionalized double-net/TA dynamic hydrogel promotes cartilage regeneration through stabilization of chondrocyte phenotype
Source: Mater Today Bio. 2025 Aug 16;34:102203. doi: 10.1016/j.mtbio.2025.102203 (PMC12395504; doi:10.1016/j.mtbio.2025.102203)
Supplement: Multimedia component 1 [file mmc1.pdf]

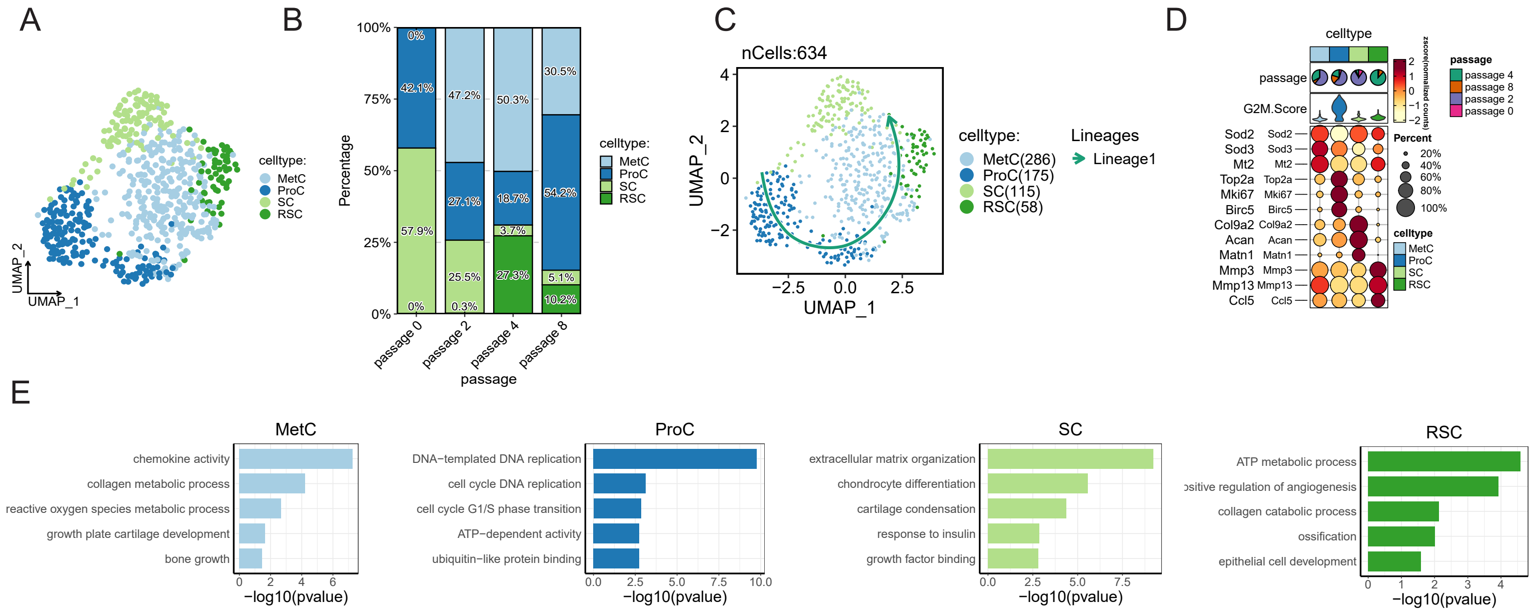

Figure S1 (A) UMAP visualization of mouse chondrocytes clustered into 4 distinct subtypes: RSCs, SCs, ProCs, and MetCs. (B) Proportion of each cell type of 4 passages of mouse chondrocytes. (C) Cell differentiation trajectory predicted by Slingshot. (D) Marker genes for each cell type. (E) GO enrichment for each cell types. (RSC: Remodeling Stromal Cell; SC: Stromal Cell; ProC: Proliferative Cell; MetC: Metabolic Cell GO: Gene Ontology. Mouse single cell data were downloaded from GEO database with accession id GSE193744.)
