# Supplementary material for: Antioxidant functionalized double-net/TA dynamic hydrogel promotes cartilage regeneration through stabilization of chondrocyte phenotype
Source: Mater Today Bio. 2025 Aug 16;34:102203. doi: 10.1016/j.mtbio.2025.102203 (PMC12395504; doi:10.1016/j.mtbio.2025.102203)
Supplement: Multimedia component 2 [file mmc2.pdf]

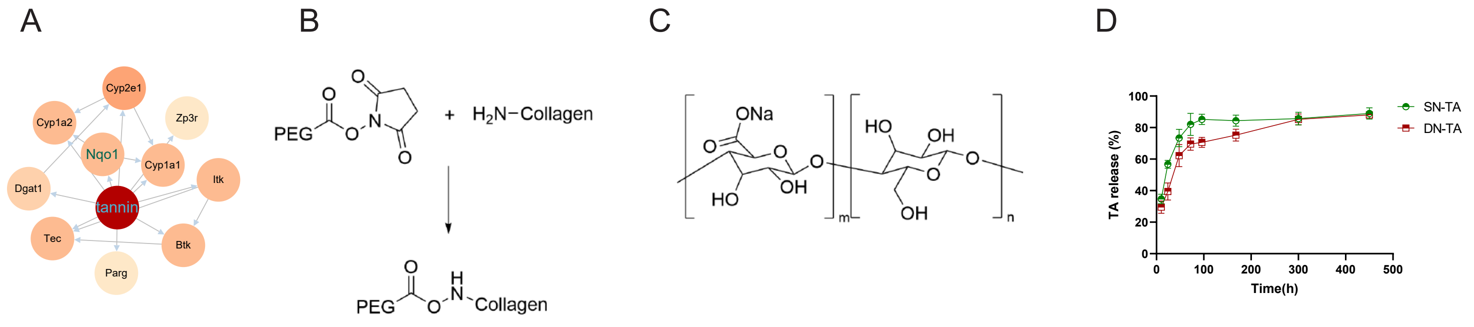

Figure S2 (A) Network of tannin-associated proteins interactions obtained from STITCH database. (B) Conjugation of PEG to collagen via an NHS ester reaction. (C) Chemical structure of CNF (cellulose nano-fibroblast). (D) TA release cureves within SN and DN group. (PEG: Polyethylene Glycol; CNF Cellulose Nano-fibroblast; TA: Tannin Acid; SN: Single Net; DN: Double Net)
