# Supplementary material for: Antioxidant functionalized double-net/TA dynamic hydrogel promotes cartilage regeneration through stabilization of chondrocyte phenotype
Source: Mater Today Bio. 2025 Aug 16;34:102203. doi: 10.1016/j.mtbio.2025.102203 (PMC12395504; doi:10.1016/j.mtbio.2025.102203)
Supplement: Multimedia component 3 [file mmc3.pdf]

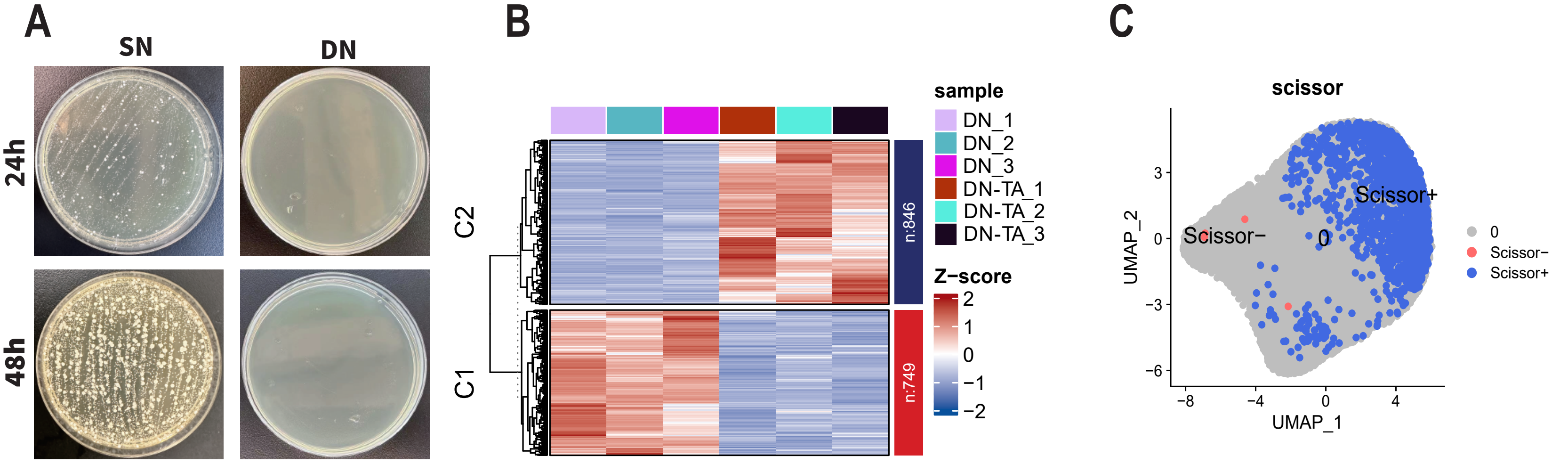

Figure S3 (A) Antibacterial activity of SN and DN materials against Staphylococcus aureus (S. aureus) over 24 and 48 hours. The DN material shows superior resistance, with minimal bacterial growth, while the SN material exhibits significant proliferation of S. aureus after 48 hours. (B) Heatmap of DEGs between DN and DN-TA. (C) Integrating bulk RNA data into single cell RNA data by R package “Scissor”. Scissor+ represented cellular phenotypes correlated to DN-TA group. This indicated DN-TA increased the cell proportion of SC. (SN: Single net; DN: Double net; DEG: Differentially expressed gene; SC: Stromal Cell.)
